# Low and moderate, rather than high intensity strength exercise induces benefit regarding plasma lipid profile

**DOI:** 10.1186/1758-5996-2-31

**Published:** 2010-05-21

**Authors:** Fabio S Lira, Alex S Yamashita, Marco C Uchida, Nelo E Zanchi, Bruno Gualano, Eivor Martins, Erico C Caperuto, Marília Seelaender

**Affiliations:** 1 Molecular Biology of the Cell Group, Institute of Biomedical Sciences, Department of Cell and Developmental Biology, University of São Paulo, Brazil; 2 Department of Physiology, Division of Nutrition Physiology, Federal University of São Paulo, São Paulo, Brazil; 3 Department of Biological Sciences and Health, UniFIEO, São Paulo, Brazil; 4 Laboratory of Applied Nutrition and Metabolism, Physical Education and Sport School, University of São Paulo, Brazil; 5 Mackenzie Presbiterian University, São Paulo, Brazil

## Abstract

**Background:**

The effects of chronic aerobic exercise upon lipid profile has been previously demonstrated, but few studies showed this effect under resistance exercise conditions.

**Objective:**

The aim of this study was to examine the effects of different resistance exercise loads on blood lipids.

**Methods:**

Thirty healthy, untrained male volunteers were allocated randomly into four groups based at different percentages of one repetition maximum (1 RM); 50%-1 RM, 75%-1 RM, 90%-1 RM, and 110%-1 RM. The total volume (sets × reps × load) of the exercise was equalized. The lipid profile (Triglycerides [TG], HDL-cholesterol [HDL-c], LDL-cholesterol, and Total cholesterol) was determined at rest and after 1, 24, 48 and 72 h of resistance exercise.

**Results:**

The 75%-1 RM group demonstrated greater TG reduction when compared to other groups (p < 0.05). Additionally, the 110%-1 RM group presented an increased TG concentration when compared to 50% and 75% groups (p = 0.01, p = 0.01, respectively). HDL-c concentration was significantly greater after resistance exercise in 50%-1 RM and 75%-1 RM when compared to 110%-1 RM group (p = 0.004 and p = 0.03, respectively). Accordingly, the 50%-1 RM group had greater HDL-c concentration than 110%-1 RM group after 48 h (p = 0.05) and 72 h (p = 0.004), respectively. Finally, The 50% group has showed lesser LDL-c concentration than 110% group after 24 h (p = 0.007). No significant difference was found in Total Cholesterol concentrations.

**Conclusion:**

These results indicate that the acute resistance exercise may induce changes in lipid profile in a specific-intensity manner. Overall, low and moderate exercise intensities appear to be promoting more benefits on lipid profile than high intensity. Long term studies should confirm these findings.

## Introduction

It is well established that physical inactivity is related to decreased high density lipoprotein cholesterol (HDL-c) and exceeded triglycerides (TG) concentrations, which contribute, at least partially, to increased atherosclerotic disease(s) risk [1]. On the other hand, chronic exercise training has favorable effects on lipid profile [2-4]. In this context, increased exercise practice, mainly continuous aerobic exercise, has been considered one of the best non-pharmacological strategies in preventing and treating cardiovascular diseases [5].

Although the effect of chronic aerobic exercise upon lipid profile has been demonstrated, few studies have shown this effect under resistance exercise conditions. Sallinen *et al*. [6] showed that older men submitted to chronic resistance training schedule by 21 weeks (80% of 1-RM), do not improved blood lipid profile, while systolic blood pressure and muscle strength was improved. The authors suggested that chronic resistance exercise improved markers of cardiovascular risk and the resistance exercise alone was not enough to induce a favorable lipid profile. A similar result was observed by Elliott *et al*. [7], were postmenopausal women, executing resistance training sessions by 16 weeks (80% of 10-RM, three times a week) did not show changes in lipid profile, although presented improved muscle strength. On the other hand, premenopausal women trained during 14 weeks (85% of 1-RM, three times a week) showed a 9% decrease in total cholesterol, 14% decrease in LDL-cholesterol, and 14.3% in the ratio total cholesterol/HDL-c.

In another study performed with untrained males comparing two different protocols (low vs. high-repetition; 5-RM and 15-RM, respectively) by 10 weeks (three times a week) Kokkinos *et al*. [8] showed no difference in lipoprotein profiles. Finally, a meta-analysis of randomized controlled trial showed that chronic resistance training decreased total cholesterol, LDL-cholesterol, TG, and increased HDL-c [9].

When the main issue is acute exercise, little is known about the acute resistance training upon blood lipid profile in healthy people. Thus, few information exist about the effects of different resistance exercise intensities on lipid profile.

The effective exercise training in lipid profile is dependent of exercise intensity, duration and frequency of each session associated with the length of the exercise training period [10]. Therefore, we hypothesized that acute resistance exercise may induce changes in lipid profile in a specific-intensity manner. We aimed to compare the effect of four different acute strength resistance intensities (50%, 75%, 90% and 110%-1 RM) on the lipid profile in healthy men.

## Methods

### Experimental design and 1 RM determination

Subjects were randomly placed into four groups; 50%-1 RM, 75%-1 RM, 90%-1 RM and 110%-1 RM based on the intensity of a bench press exercise. One repetition maximum (1 RM) of the bench press was determined seven days prior to the exercise bout for all subjects. For the determination of 1 RM, subjects were instructed to grip the bar at a comfortable position with was typically 10-20 cm wider than the shoulder width^8^, and performed a warm-up consisting of 8-10 repetitions using a light weight, 3-5 repetitions using a moderate weight, and 1-3 repetitions using a heavy weight. After the warm-up, each subject was tested for 1 RM strength by increasing the resistance on subsequent attempts until the subject was unable to complete an attempt. Each attempt was separated by 3-5 minutes of rest [11] and two trained spotters were always present. The criterion measures consisted of lipid profile (Triglycerides, HDL-cholesterol, Total Cholesterol, and LDL-cholesterol). Theses measurements were taken before exercise, and 1, 24, 48 and 72 h following exercise, and changes in the measures over time were compared amongst the groups.

### Participants

Experimental sequence has been described previously by Uchida et al. [12]. Thirty Brazilian Army male soldiers (mean ± standard deviation: age, 19.1 ± 1.8 years; 176.9 ± 6.5 cm; body mass, 70.9 ± 8.1 kg; body mass index, 26.6 ± 2.8 kg/m^2^) volunteered to participate in this study. They were randomly placed into one of the five groups, and the number of participants for each group was 50%-1 RM (n = 8), 75%-1 RM (n = 8), 90%-1 RM (n = 7), and 110%-1 RM (n = 7). No significant differences in the age, height, body weight, and 1 RM strength existed amongst the groups [12]. The participants had involved in a military physical training (running for 60 min and circuit training with body weight exercises for 30 min) 3 sessions per week before this study. They were experienced in resistance training exercises including the bench press exercise for a minimum of one year. However, all of them had not been involving in resistance training for more than one year prior to this study. Participants were free from any musculoskeletal or neurological problems. All participants were instructed to refrain from any strenuous activities 72 h before and during the experimental period.

Finally, this study was approved by the institutional ethics committee and all participants were informed of the purpose and risk of the study before signing an informed consent form.

### Exercise protocol

The participants in the 50%-1 RM, 75%-1 RM, 90%-1 RM and 110%-1 RM groups performed a bench press warm-up routine (2 sets of 12 repetitions at 30%-1 RM, resting for 2 minutes between sets) before the exercise bout. In the bench press bout, the 50%-1 RM group performed 4 sets of ~ 20 repetitions, the 75%-1 RM group performed 5 sets of ~ 11 repetitions maximum, the 90%-1 RM group performed 10 sets of 4 repetitions maximum, and the 110%-1 RM group performed 8 sets of ~ 4 repetitions maximum, based on the 1 RM determined previously. The participants performed each set with maximal effort until they were unable to perform the repletion with proper technique, which was judged by the investigator. Thus the number of repetition was not exactly the number shown above. In the 110%-1 RM condition, subjects performed the eccentric phase only with the load, and during the concentric phase, two spotters raised the starting position. The rest between sets was 2 min for all conditions. The participants in the 50%-1 RM, 75%-1 RM and 90%-1 RM groups were instructed to move the bar 2 s for the eccentric phase and 1 s for the concentric phase, and to lower the bar to the point where touching the chest immediately followed by a full arm extension. The participants in the 110%-1 RM group were asked to spend 3 s for eccentric contraction for each repetition. The total volume of each participation was calculated by the formula: Total Volume (kg) = Number of sets × Number of repetitions × load (kg) [12,13].

### Blood sampling and analyses

The blood samples (10 ml) were obtained from the antecubital vein in the morning (07:00-09:00 am) after 12 h overnight fasting period before and 1, 24, 48 and 72 h after exercise. The blood was immediately put into two 5-ml vacutainer tubes (Becton Dickinson, BD, Juiz de Fora, MG, Brazil) containing EDTA for plasma separation. The tubes were centrifuged at 250 g for 15 minutes at 4°C, and plasma samples were stored at -80°C until analysis. Triglycerides, HDL-cholesterol, Total cholesterol were assessed trough commercial enzymatic kits (Labtest^®^, São Paulo, Brazil). LDL-cholesterol was calculated according to Friedewald et al. [14].

### Statistical analyses

SAS^® ^proc Mixed was used to analyze repeated measures and, when applicable, Tukey Post hoc was used for multiple comparisons. The groups (50%-1 RM, 75%-1 RM, 90%-1 RM, and 110%-1 RM) and periods (rest, 1, 24, 48 and 72 h) were considered fixed factors and the subjects were assigned as random factors. The degrees of freedom were adjusted by proc Kenward-Roger method.

For baseline comparisons between the groups two-sample independent t-test was used. All data is expressed as mean ± SD. The significance level adopted to reject the null hypothesis was p ≤ 0.05.

## Results

All subjects in the exercise group performed maximal repetitions for each set as instructed, and the number of repetitions was close to the expected number for each intensity (~20 for 50% 1-RM; ~11 for 75% 1-RM; ~4 for 90% 1-RM; ~4 for 110% 1-RM). Although the number of repetitions decreased with increasing the number of set, no significant difference in the total volume of the exercise amongst the groups was observed (p = 0.1) as described previously by Uchida et al. [12].

**Figure 1:**
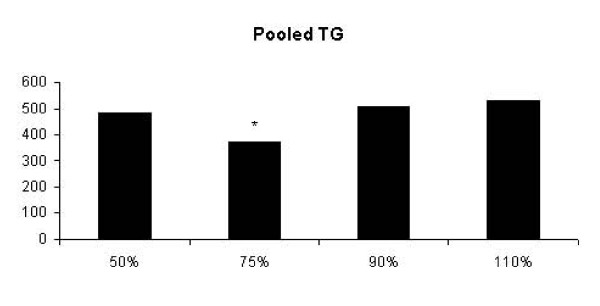
**Pooled TG data (1 h + 24 h + 48 h + 72 h) of the 50%-1 RM, 75%-1 RM, 90%-1 RM and 110%-1 RM groups**. The TG concentration in 75%-1 RM group was decreased when compared to other groups (* p < 0.05).

The Mixed Model statistic analysis showed a significant mean group effects for TG levels (p = 0.02). For this reason, we expressed TG levels in pooled data (1 h + 24 h + 48 h + 72 h) (Figure 1). It was noted that acute resistance exercise at 75% 1-RM decreased TG levels when compared to 50% 1-RM, 90% 1-RM and 110% 1-RM groups (p = 0.03, p = 0.02, p = 0.003, respectively). Additionally, it was observed significant interaction effects (group × time) for TG levels at 72 h after 75% 1-RM exercise bout (Table 1). The 50% 1-RM and 75% 1-RM groups decreased (p = 0.01, p = 0.01, respectively) TG levels, when compared with 110% -1 RM group in response to exercise bout. At the same time, 75% 1-RM group decreased TG levels when compared to 90% group (p = 0.03) 72 h after the exercise bout.

**Table 1:** Lipoprotein profile in 50%-1 RM, 75%-1 RM, 90%-1 RM and 110%-1 RM groups.

	Groups	Rest	1 h	24 h	48 h	72 h
*TG* *(mg.dL^-1^)*	50% 1-RM	100.5 ± 32.3	96.7 ± 29.0	100.5 ± 42.7	101.0 ± 33.8	85.9 ± 25.0**
	75% - 1 RM	79.3 ± 20.6	69.0 ± 17.7	75.9 ± 24.3	80.6 ± 21.6	68.3 ± 21.9**^#^
	90% - 1 RM	102.1 ± 19.8	87.0 ± 14.8	98.8 ± 27.3	87.8 ± 25.0	111.6 ± 78.6
	110% - 1 RM	115.3 ± 47.2	102.1 ± 25.5	89.8 ± 23.1	113.6 ± 54.7	127.4 ± 77.2
						
*Total cholesterol* *(mg.dL^-1^)*	50% 1-RM	122.7 ± 27.4	128.6 ± 34.6	116.9 ± 39.8	130.5 ± 31.1	139.2 ± 33.3
	75% - 1 RM	110.3 ± 24.7	120.5 ± 21.9	112.2 ± 19.0	112.3 ± 16.4	111.7 ± 22.6
	90% - 1 RM	124.5 ± 19.9	126.4 ± 25.3	127.4 ± 22.6	123.5 ± 25.3	123.3 ± 20.6
	110% - 1 RM	126.7 ± 18.7	128.0 ± 24.7	126.8 ± 20.9	130.1 ± 22.3	125.5 ± 15.5
						
*LDL-c* *(mg.dL^-1^)*	50% 1-RM	67.5 ± 7.9	62.4 ± 30.6	51.0 ± 36.5*	86.8 ± 56.7	83.8 ± 31.3
	75% - 1 RM	55.2 ± 32.3	78.2 ± 26.2	58.2 ± 19.5	52.9 ± 7.8	75.6 ± 32.6
	90% - 1 RM	55.3 ± 34.1	73.6 ± 32.9	69.2 ± 33.1	76.5 ± 21.6	66.1 ± 37.2
	110% - 1 RM	85.4 ± 26.4	81.1 ± 25.2	84.1 ± 18.9	84.5 ± 31.3	73.8 ± 19.7
						
*HDL-c* *(mg.dL^-1^)*	50% 1-RM	41.8 ± 7.1	48.8 ± 10.6	45.7 ± 14.7	40.6 ± 8.7**	41.9 ± 16.9*
	75% - 1 RM	38.4 ± 15.8	32.6 ± 19.5	40.5 ± 16.9	40.6 ± 8.8*	36.3 ± 18.8
	90% - 1 RM	45.7 ± 25.4	39.9 ± 16.1	37.0 ± 13.6	26.1 ± 5.8	30.2 ± 12.6
	110% - 1 RM	27.4 ± 10.3	33.5 ± 4.9	29.6 ± 3.0	26.6 ± 7.0	27.7 ± 3.7

TG, Total Cholesterol, LDL-c and HDL-c concentration in 50%-1 RM, 75%-1 RM, 90%-1 RM and 110%-1 RM groups. The 50% 1-RM and 75% 1-RM group showed a decreased in TG concentration when compared with 110% 1-RM group 72 h after the exercise bout (**P = 0.01 vs 110% 1-RM). At the same time, 75% 1-RM group decreased TG concentration when compared whit 90% 1-RM group (^#^P < 0.05 vs 90% 1-RM). In relation LDL-c, the 50% group has showed lesser LDL-c concentration than 110% group after 24 h (*P < 0.05 vs 110% 1-RM). HDL-c was increased in 50% 1-RM and 75% 1-RM group when compared whit 110% 1-RM 48 hours after the exercise bout (*P < 0.05 vs 110% 1-RM; **P < 0.01 vs 110% 1-RM).

**Figure 2:**
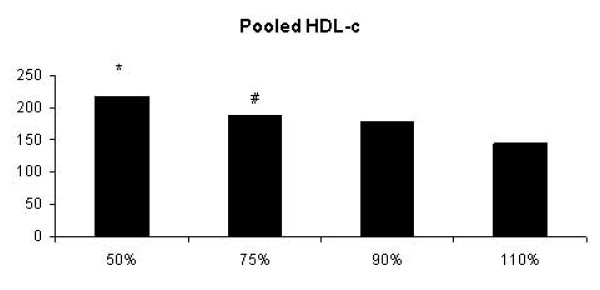
**Pooled HDL-cholesterol data (1 h + 24 h + 48 h + 72 h) of the 50%-1 RM, 75%-1 RM, 90%-1 RM and 110%-1 RM groups**. HDL-c concentration was significantly greater after strength exercise in 50%-1 RM and 75%-1 RM groups when compared to 110%-1 RM group (* p = 0.004 and # p = 0.03, respectively).

It was verified significant mean group effect for HDL-c (p = 0.005). Then, we also expressed these results as polled data (Figure 2). HDL-c levels was significantly increased in 50% 1-RM and 75% 1-RM groups when compared to 110% 1-RM group after 48 hours (p = 0.004 and p = 0.03, respectively). Moreover, it was showed a significant interaction effects after 72 h in 50% 1-RM group for HDL-c levels when compared to 110% 1-RM group (Table 1).

Significant interaction effects were also observed for LDL-c (Table 1). Twenty-four hours after strength exercise, the 50% 1-RM group has showed a decreased LDL-c levels when compared with 110% 1-RM group. Furthermore, the 50% 1-RM group presented a time-effect increase in LDL-c concentration from 48 h (p = 0.05). The 75% group had a significant LDL-c increase after 1 h (p = 0.04), which was reduced to baseline levels after this point. No significant differences were observed for Total Cholesterol (Table 1).

## Discussion

The present investigation examined four different intensities of an acute resistance exercise protocol on the blood lipid profile at rest and after 1, 24, 48 and 72 h. Endurance exercise-induced changes in HDL-c is the resulting interaction between exercise intensity, duration and frequency of each session coupled with the length of the exercise training period [13]. Both, acute and chronic effects of endurance exercise on the lipoprotein metabolism have been extensively documented and reviewed [15,16]. On the other hand, the effects of resistance exercise on the lipoprotein concentrations remain equivocal.

We observed that the moderate resistance exercised group (75%-1 RM) reduced the TG compared to 50%, 90% and 110% intensities. Interestingly, the low and moderate resistance exercised group (50% and 75%) increased HDL-c when compared to the high intensity group (110%) and no significant differences in LDL-cholesterol and total cholesterol concentrations were observed. Collectively, these results suggest by the first time, for our knowledge, that there may be a dose-response relationship between resistance training intensity and lipid profile modulation.

The effect of resistance training upon blood lipid profile has been shown to favorably modify the lipoproteins [17,18], while others have reported no changes [6-8]. For example, Wallace et al. [7] comparing acute high volume (10-12 RM) vs. acute low volume (1-5 RM loads) exercise showed, in healthy trained males, an increased in HDL-c 24 h after acute high volume exercise bout, without changes in total cholesterol and TG. However, this study presented an important bias, due to the equalization absence in the work produced by the exercise bouts (sets × repetitions × weight). Therefore, it is possible that the different volumes of training used in the supra-cited study could have contributed to the differences in the results obtained. Hill et al. [19] showed that acute high intensity (10-RM) vs. low intensity (5-RM) does not modulate TG, total cholesterol and LDL-c, and only high intensity resistance exercise increased HDL-c immediately after the exercise bout. The authors suggested that HDL-c response is intensity-dependent, supporting the concept that an adaptive threshold may exist in resistance exercise.

The factors modulating the lipid profile in chronic resistance exercise remain still unclear. However, although decreased body mass and body fat have also been associated with decreased total cholesterol, LDL-cholesterol and increased HDL-c, longitudinal studies employing chronic resistance training were not related with body fat and lipid profile changes [8,19].

Another possible mechanism modulating the HDL-c is the pathway of reverse cholesterol [1]. This route removes cholesterol from the circulation and distributes it to the peripheral tissues and the liver. Basically this mechanism follow the principle that during acute and chronic aerobic exercise, lecithin activity cholesterol aciltransferase (L-CAT), the enzyme responsible for the cholesterol ester transfer to the HDL-c is increased, and plasmatic cholesterol ester transfer protein (CETP) activity is reduced (the enzyme responsible for the transfer of ester of HDL cholesterol to the other lipoproteins) [2,20,21]. In fact, this hypothesis, which is now accepted in endurance exercise training, can be applied in acute resistance training. Although Wallace et al. [17] did not equalized the total exercise volume, they showed an increased in 27% in L-CAT activity in the high volume group 24 h after the exercise bout. Thus, it is possible to speculate that decreased concentrations of total cholesterol in the plasma might be attained through the exchange of cholesterol ester between tissues and lipoproteins to the HDL-c [1]. However, the precise mechanisms by which low (50% 1-RM) and moderate (75% 1-RM) resistance exercise intensities modulate the concentration of HDL-c needs to be better examined.

In our study acute moderate bench press resistance exercise (75%) reduced pooled TG concentrations when compared with other intensities. Magkos et al [22] attributed possible routes of VLDL and TG removal from plasma, including hydrolysis by lipoprotein lipase (LPL) and possibly also by hepatic lipase, transfer of TG to other lipoproteins, conversion of VLDL to lipoproteins of higher density, i.g., intermediate-and low-density lipoproteins (IDL and LDL, respectively), as well as removal of the whole VLDL particle from plasma via interaction with hepatic and/or peripheral receptors [22-24]. Furthermore, it is well documented that regular physical exercise is able to induce the augment of LPL gene expression and activity in skeletal muscle [25] resulting in decreased plasma TG content, which is also linked with decreased liver VLDL output [4,26]. A relationship between the increased catabolic rate of TG during the early phase of recovery and repletion of the intramuscular TG pool is further suggested by the transient increase in the transcription rate of muscle LPL as described by Pilegaard et al. [27]. As a potential alternative explanation, Tsekouras et al. [24] showed that acute resistance exercise increased the clearance of the VLDL and TG, and decreased the mean residence time of such lipoproteins in the circulation, thus contributing to decreased lipoprotein levels. Finally, Magkos et al. [22] observed that acute resistance exercise was efficient in order to increase the clearance of VLDL and TG, and decrease the mean residence time of such lipoproteins in the circulation, suggesting a particular regulatory mechanism elicited by acute resistance exercise.

In the present study, it was observed a time-course effect of acute strength exercise on lipid profile, which is in line with previous aerobic exercise findings [23,28-30]. Such changes may be related to the aforementionated mechanisms. Also, we cannot rule out the possibility that other factors may contribute (or potentialize) to these findings, such as changes hormonal concentration in plasma, muscle LPL activity, sympathoadrenal activity, and CETP activity. In summary, the present study revealed that low/moderate intensity acute resistance exercise (≤ 75% 1 RM) is capable to promote more beneficial changes on lipid profile than high intensity acute resistance exercise.

## Competing interests

The authors declare that they have no competing interests.

## Authors' contributions

FSL helped in the treatment, study design, carried out some biochemical analyses and participated in manuscript preparation, ASY helped in the treatment, study design, carried out some biochemical analyses and participated in manuscript preparation, MCU helped in the treatment and participated in manuscript preparation, NEZ helped with lipid profile analysis and critical revision of manuscript, BG performed all statistics analysis, EMJr helped in the treatment and participated in manuscript preparation, ECC helped with lipid profile analysis and critical revision of manuscript, MCLS supervises the study and helped in manuscript preparation. All the authors have read and approved the final manuscript.
